# Mapping evidence on health promotion in HIV testing among men who have sex with men and transgender women using the social-ecological model and the vulnerability theoretical framework: a scoping review

**DOI:** 10.1186/s12889-023-16860-9

**Published:** 2023-10-07

**Authors:** Camila Amaral Moreno Freitas, Thais Aranha Rossi, Inês Dourado, Marcelo Eduardo Pfeiffer Castellanos, Nathalia Sernizon Guimarães, Laio Magno

**Affiliations:** 1 Instituto de Saúde Coletiva, Universidade Federal da Bahia (UFBA), Basílio da Gama Street, Salvador, BA 40110-040 Brazil; 2 Departamento de Ciências da Vida, Universidade do Estado da Bahia (UNEB), 2555 Silveira Martins Street, Salvador, BA 41150000 Brazil

**Keywords:** Testing barriers, Testing facilitators, Health promotion, HIV testing, Community approaches, Social vulnerability, Socio-ecological model

## Abstract

This study aimed to map the scientific evidence on health promotion in human immunodeficiency virus) HIV testing among men who have sex with men (MSM) and transgender women (TGW) based on the social-ecological model (i.e., individual, organizational and social levels) and the theoretical framework of vulnerability (i.e., individual, social, and programmatic levels). The reviewed studies indicated several barriers to accessing HIV testing (e.g., economic, structural, and bureaucratic) and demonstrated the potential for community approaches to promote greater access to HIV testing and minimize the stigma and discrimination associated with HIV testing, primarily through community leadership and social support networks. The socio-ecological model of health promotion and the vulnerability approach have the potential to contribute to improving HIV testing services by balancing the technical and political power of health services and providers with community participation while considering the social contexts. Therefore, there is a need for reflection on health promotion policies and programs aimed at expanding access to HIV testing among MSM and TGW through interventions that consider the social contexts and cultural perspectives. Moreover, inter-sectoral strategies aimed at improving living conditions and access to fundamental resources for maintaining health and well-being should be considered.

**Supplementary Information:**

The online version contains supplementary material available at 10.1186/s12889-023-16860-9.

## Strengths and limitations

As this is a scoping review, this article maps the scientific production of health promotion in HIV testing that, added to our theoretical framework, indicates that health promotion, combined with HIV testing in community strategies, makes access to HIV testing more democratic. As this is a broad review, some aspects of testing related to health promotion deserve to be further systematically investigated.

## Background

In 2022, approximately 6 million people worldwide living with HIV (PLHIV) were unaware of their serological status [1]. The number of human immunodeficiency virus (HIV) tests performed decreased worldwide between 2020 and 2021 due to the novel coronavirus pandemic. For example, Latin America and the Caribbean had approximately 4,000 fewer diagnoses, and in eastern and southern Africa, approximately 10,000 fewer HIV tests were conducted in 2020 and 2021 than in 2019. European Union (EU) countries reported declining HIV tests performed during 2020 [2]. The pandemic notably increased social inequalities aggravated social vulnerability, and created barriers to accessing various health services, substantially affecting historically impoverished and stigmatized populations [3].

The absence of HIV testing represents a significant challenge in controlling the epidemic, as knowledge of the serological status for HIV detection is the gateway to healthcare, including initiating anti-retroviral therapy (ART) [2, 4–7]. ART significantly helps prevent viral transmission because PLHIV on ART with an undetectable viral load do not transmit the virus to others; the so-called “undetectable = untransmissible” [8].

The current UNAIDS targets 95–95-95 to eliminate the HIV epidemic by the year 2025 are as follows: “95% of people living with HIV know their serological status; 95% of people who know their serological status are under anti-retroviral treatment; and 95% of people undergoing anti-retroviral treatment have their viral load suppressed” [9]. However, these will only be achieved with increased HIV testing and awareness of one’s serological status.

Representing 65% of global HIV infections, men who have sex with men (MSM) and transgender women (TGW) are considered more vulnerable to the HIV epidemic [9, 10]. Thus, they have a greater need for HIV testing [11–13]. Studies have shown that the testing frequency is low in this population in some parts of the world [14–16]. Barriers to expanding test coverage include a low perception of risk, fear of positive testing, concerns about confidentiality and stigma, inconvenience of attending clinics, and long waiting times [17–20].

In several countries, testing strategies for MSM and TGW are still rooted in a biomedical model, with health professionals’ focus restricted to the technological dimension of preventive practices (e.g., testing in health services) and prescription of anti-retroviral drugs such as post- (PEP) and pre- (PrEP) exposure prophylaxis [18, 21, 22]. Generally, these interventions are guided by technical and bureaucratic views, with little flexibility to adapt to socio-cultural perspectives, such as government imposition of programs and funding policies that prevent hiring peer counselors in specific contexts [16, 21]. Furthermore, there are numerous records of discriminatory practices inside and outside HIV testing services that make accessing them difficult, such as discriminatory attitudes among healthcare service staff or a lack of privacy and confidentiality concerning a patient’s serologic status [18, 21, 23, 24].

Therefore, it is necessary to reflect on health promotion policies and programs to expand access to HIV testing among MSM and the TGW through interventions that consider key populations' social contexts and cultural perspectives [16]. Moreover, inter-sectoral strategies aimed at improving living conditions and access to fundamental resources for maintaining health and well-being should be considered under the auspices of equity and in accordance with the approaches proposed by the International Conferences on Health Promotion [25].

Accordingly, through this scoping review, we aimed to map the scientific evidence on health promotion in HIV testing among MSM and TGW based on the concept of the social-ecological model, which establishes three explanatory levels: (i) the individual level (e.g., individual characteristics such as education, habits and lifestyle, risk perception, and personal beliefs); (ii) the organizational level (e.g., perceptions of control over the environment such as cultural, organizational, and geographical dimensions of services); and (iii) the social level (e.g., community approaches to health promotion and development of well-being [26]. Stokols (1996) point out that these levels address elements that can assist in developing and evaluating health promotion programs. In addition, we employed the theoretical framework of vulnerability [27] to better understand how the inter-relationships among the individual, organizational, and social levels lead to different outcomes in HIV testing. By bringing together the vulnerability framework and social-ecological model of health promotion, we can acquire an all-encompassing view of the planning of actions at these three equivalent levels, which may help overcome existing limitations in implementing health promotion policies for HIV testing [28].

## Materials and methods

This study adopted a scoping review described according to the recommendations of the PRISMA Extension for Scoping Reviews (PRISMA-ScR): Checklist and Explanation [29] on HIV testing among MSM and TGW from a social-ecological approach to health promotion, combined with the theoretical framework of vulnerability (The Checklist for Scoping View is attached as a related file). This study was registered on the Open Science Framework platform (https://osf.io/ys359/).

### Eligibility criteria

**Fig. 1:**
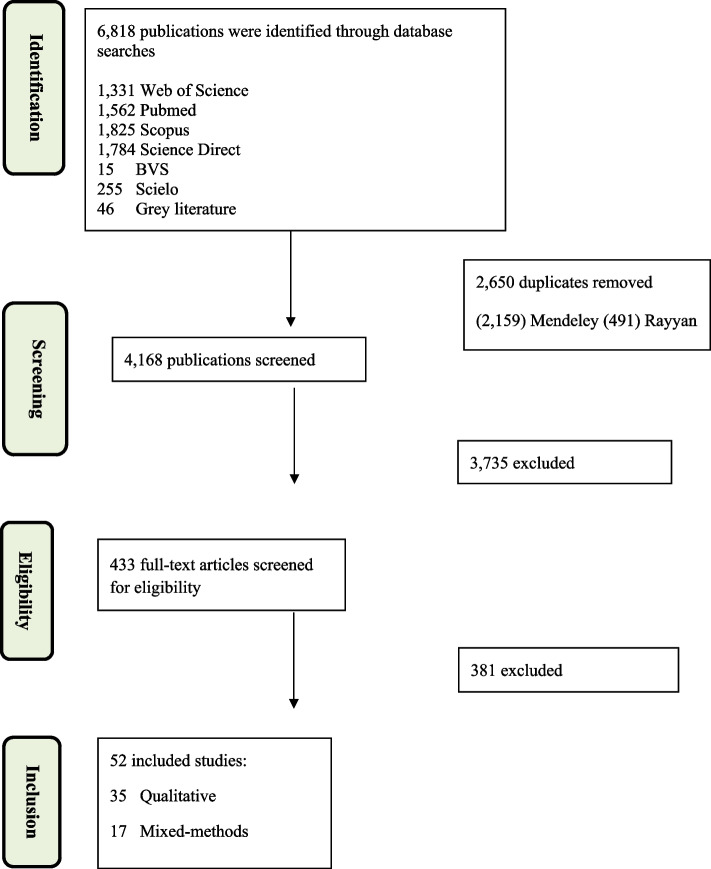
Flowchart for the systematic article selection process

Qualitative and mixed methods studies that described health promotion in HIV testing among MSM and TGW based on the conception of the social-ecological model were included. The data collection of these studies was performed using focus groups and in-depth or semi-structured interviews to evaluate HIV testing among MSM and TGW. Abstracts presented at congresses were also included. We excluded narrative, integrative, scoping, rapid or systematic reviews; studies that do not report HIV testing on MSM or TGW; and protocols. No restrictions were imposed on the dates or places of publication (see Fig. 1).

### Data source

The search for information was conducted using the electronic databases of the Web of Science, MEDLINE, PubMed, Scopus, Science Direct, Scielo (via the Virtual Health Library), and gray literature. The review was conducted between June 2020 and updated in December 2022.

The list of terms identified in MeSH (medical subject headings) or DeCS (health sciences descriptors) used to search for articles was as follows: “sexual and gender minorities,” “trans people,” “transvestism,” “men who have sex with men,” “rapid test,” “testing strategies,” “anonymous tests,” “serological tests,” “HIV serodiagnosis,” “AIDS serodiagnosis,” “early diagnosis,” “HIV infection,” “HIV infection/diagnosis,” and “HIV.” The information search strategy included combining the descriptors and using Boolean indicators “OR” and “AND.” The correspondence between Portuguese and Spanish was also used. Furthermore, a manual search was performed on all the included study reference lists to identify potential local studies.

### Selection of studies and data extraction

The studies were managed using Mendeley to remove duplicates and were subsequently exported to the Rayyan Qatar Computing Research Institute® platform to assist the researchers in the eligibility screening process. Two authors independently identified and verified the titles and abstracts of the studies and then performed textual evaluations. In cases of disagreement, a third author resolved the conflicts.

The research team prepared and applied a data extraction spreadsheet (see Supplementary Material 1) to summarize the following data from the studies: reference (name and year of publication of the study) study location (country), title, journal, objectives, population studied, methodology, study scope in relation to the population (MSM and TGW), type of testing creation strategy request, HIV prevalence, barriers and facilitators for HIV testing, main results, and final considerations.

A meta-ethnographic approach comprising six phases was adopted to analyze the articles [30]: 1) definition of the theme and scope of the study; 2) choice of relevant studies through eligibility criteria and evaluation of methodological quality using the Critical Appraisal Skills Program (CASP) score; 3) careful reading of the articles and extraction of primary data or first order constructs (untreated data resulting from interviews, focus groups, or research in general); 4) analyzing the key concepts of the articles and examining these concepts’ relationships between studies, extracting the concepts from second order constructs (authors’ primary interpretation of the data) and from study themes; 5) producing the constructs from the analysis and comparison of the studies’ key concepts, seeking to identify the presence or absence of similarities, and grouping those that stood out or were repeated into categories; and 6) producing a content synthesis, which can be a refutational synthesis or a line of argument synthesis [30, 31].

### Theoretical framework

The theoretical framework of vulnerability postulates the existence of a “set of aspects that are not only individual but collective and contextual, leading to greater susceptibility to infection and illness and, inseparably, the greater or lesser availability of resources of all kinds to protect against both”. Vulnerability analyses consider three interconnected dimensions: individual, programmatic, and social [27].

The socio-ecological model framework proposes an interface among social ecology, behavioral medicine, and public health. It is comprised of theoretical principles that aim to clarify the inter-relationships between individual and environmental factors and their interference health outcomes [26]. It offers a variety of methodological concepts and tools for organizing and evaluating health interventions and promotions. Stokols [26] presents three levels of complementary perspectives that could generate analytical categories for health promotion interventions: 1) individual characteristics and behavioral and lifestyle changes (individual level); 2) perceptions of control over the environment, forms of organization of the environment, services, and health systems (organizational level); and 3) socio-ecological analyses of health promotion and community approaches (social level).

**Fig. 2:**
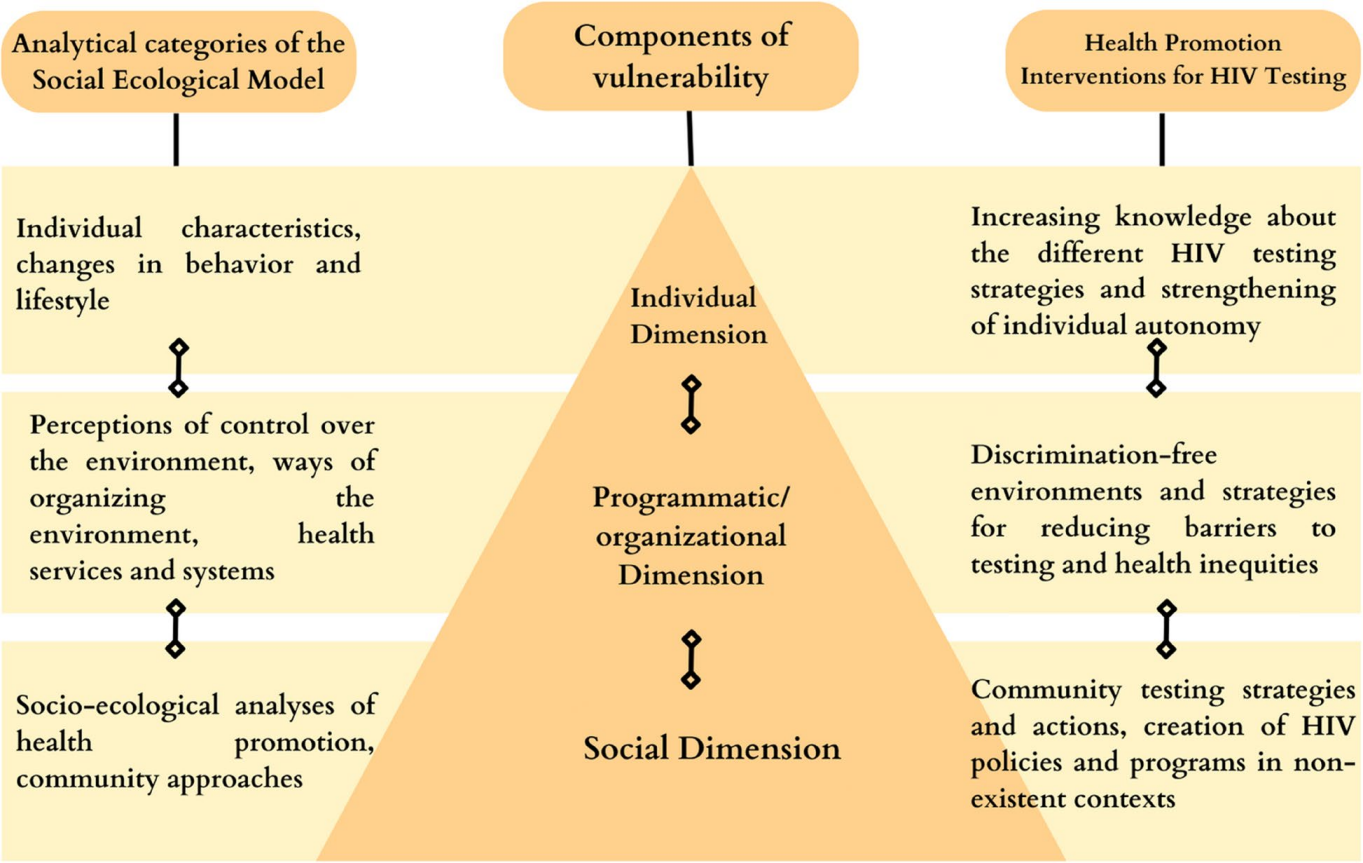
Theoretical model of health promotion related to aspects of HIV testing among MSM and TGW

Figure 2 links the elements of the theoretical vulnerability framework to the socio-ecological model to understand the conflicts, factors, and interventions that interfere with the barriers and facilitators of HIV testing among MSM and TGW. In this sense, we sought to analyze the relationships between the components of vulnerability (i.e., individual, programmatic, and social) and the socio-ecological levels (i.e., individual, organizational, and social) categorized as interconnected in the three dimensions of individual, programmatic/organizational, and social dimensions and represented as a triangle. The triangle's base represents society and community, the middle health services, and the top individual characteristics, behaviors, and relationships. These dimensions emphasize that individual health care is not only the result of individual actions but also due to a set of inter-relationships that exist among individuals, society, culture, and health institutions [26, 27, 32].

Access to HIV testing responds to social determinants that extend beyond individual dimensions. Thus, it is necessary to adopt a reflective theoretical–methodological approach to identify and analyze the problem in its dynamic totality and to identify and analyze the intersections between the vulnerability contents and structural levels. The left side of the triangle represents the main theoretical perspectives associated with determining health, disease, and care, while the right side shows the main interventions for health promotion in HIV testing (see Fig. 2).

As the individual dimension comprises strategies related to behavioral change for health promotion, it is essential necessary to consider both the quantity and quality of information that individuals possess about the problem, how they can elaborate on this information and incorporate it into their daily repertoire of concerns, and the individual’s interests and real possibilities of them transforming these concerns into safe and protective practices. Associated theoretical perspectives, such as the theories of “self-efficacy” and “risk perception” contribute to approaches to healthcare [26, 27].

The programmatic/organizational dimension addresses the strategies of environmental and organizational change for health promotion, with theoretical perspectives on organizational development and the quality of the relationship between individuals and accessed services. The environments must be free of discrimination related to 1) HIV, 2) sexual orientation, and 3) gender identity. Moreover, greater accessibility to distinct types of HIV tests is needed. In this sense, the programmatic contents of vulnerability discuss the need to guarantee public and institutional policies and ensure that social resources are made available democratically so that individuals can protect themselves from exposure to HIV and minimize its damage [26, 27].

The social dimension addresses adopting cultural change and creating models for strengthening social and community support for healthcare. In this sense, the social component of vulnerability measures the political, economic, cultural, religious, and moral contexts in which an individual is located. Specifically, it postulates the need for access to information, incorporation of this information, and the ability to transform it into practical changes. This component highlights that the ability to manage information depends on the individual and is linked to their access to education (schooling), media, and material resources [26, 27].

### Data analysis

The data analysis was based on the dimensions constructed and presented in the theoretical model (see Fig. 2). The categories were related to the social-ecological model, including HIV and auto-immunodeficiency syndrome (AIDS) policies and programs. The dimensions of vulnerability were problematized: the individual dimension: individual perceptions about HIV testing; the organizational/programmatic dimension: bureaucratization of HIV testing; and the social dimension: community strategies for HIV testing.

### Evaluation of methodological quality

The methodological quality of the primary articles was assessed using CASP [33]. The studies were classified into two categories: 1) those with high methodological rigor that complied with 9 out of 10 items and 2) those with moderate methodological rigor that complied with at least 5 of the 10 items. Validation of the article classification was discussed among the researchers [33].

## Results

### Overall characteristics

We identified 6,818 publications in the databases; 433 were screened for eligibility, and 52 were included in this study (Fig. 1). Of these, 67.3% (*n* = 35) used only a qualitative method, and 32.7% (*n* = 17) used mixed methods (i.e., qualitative and quantitative methods). Most studies (65.4%, *n* = 34) exclusively involved MSM, 30.8% (*n* = 16) concerned MSM and TGW, and 3.8% (*n* = 2) only addressed TGW. Regarding geographic distribution, most studies focused on North America, with all these articles (40%; *n* = 21) involving the United States of America (USA). Regarding the year of publication of the studies, most were published in 2018 (21.2%; *n* = 11) and 2019 (17.3%; *n* = 9) (Table 1).

**Table 1:** General characterization of the articles according to author, year, country, title, journal, and methods used

1	Authors	Home country	Year	Title	Periodic	Methods	E-score CASP
1	Fauk et al.	Indonesia	2018	The intention of men who have sex with men to participate in voluntary counseling and HIV testing and access free condoms in Indonesia	American Journal of Men’s Health	Qualitative	10
2	Dass et al.	The Netherlands	2019	Reducing health disparities: key factors for successful implementation of social network testing with HIV self-tests among men who have sex with men with a non-western migration background in the Netherlands	AIDS and Care	Qualitative	10
3	Liu F., et al.	China	2019	HIV self-testing among men who have sex with men in China: a qualitative implementation research study	Journal of Virus Eradication	Qualitative	10
4	Gohil, et al.	USA	2020	Is the Philippines ready for HIV self-testing?	BMC Public Health	Qualitative	10
5	Nanin et al.	USA	2019	HIV testing among Black and Hispanic/Latino men who have sex with men in New York City: a mixed methods study	Archives of Sexual Behavior	Mixed-method	10
6	Beattie et al.	India	2012	Personal, interpersonal and structural challenges to accessing HIV testing, treatment and care services among female sex workers, men who have sex with men and transgenders in Karnataka state, south India	Journal of Epidemiology and Community Health	Qualitative	09
7	Bilardi et al.	Australia	2013	gay and bisexual men’s views on rapid self-testing for HIV	AIDS and Behavior	Qualitative	09
8	Frye et al.	USA	2015	‘‘Just because it’s out there, people aren’t going to use it.’’ HIV self-testing among young, Black men who have sex with men and transgender women	AIDS Patient Care And STDs	Qualitative	09
9	Frye et al.	USA	2018	Preferences for HIV test characteristics among young, Black men who have sex with men and transgender women: implications for consistent HIV testing	Plos One	Qualitative	09
10	Medline E., et al.	USA	2017	HIV testing preferences among men who have sex with men members of a lesbian-gay-bi-transgender community organization in Los Angeles	Journal of the Association of Nurses in AIDS Care	Qualitative	09
11	Navaza B., et al.	Spain	2016	Provider-initiated HIV testing for migrants in Spain: a qualitative study with health care workers and foreign-born sexual minorities	PLoS ONE	Qualitative	09
12	Okoboy S., et al.	Africa	2019	Acceptability, perceived reliability and challenges associated with distributing HIV self-test kits to young men who have sex with men in Uganda: a qualitative study	Journal of the International AIDS Society	Qualitative	09
13	Wray, T, et al.	USA	2017	eTEST: developing a smart home HIV testing kit that enables active, real-time follow-up and referral after testing	JMIR Mhealth Uhealth	Qualitative	09
14	Boydell N, Buston K, McDaid, LM	UK	2017	Patterns of HIV testing practices among young gay and bisexual men living in Scotland: a qualitative study	BMC Public Health	Qualitative	09
15	Wirtz et al.	Myanmar	2017	New HIV testing technologies in the context of a concentrated epidemic and evolving HIV prevention: qualitative research on HIV self-testing among men who have sex with men and transgender women in Yangon, Myanmar	Journal of the International AIDS Society	Qualitative	09
16	Witzel et al.	UK	2016	HIV self-testing among men who have sex with men in the UK: a qualitative study of barriers and facilitators, intervention preferences and perceived impacts	Plos One	Qualitative	09
17	Witzel et al.	UK	2019	HIV self-testing intervention experiences and kit usability: results from a qualitative study among men who have sex with men in the SELPHI (Self-Testing Public Health Intervention) randomized controlled trial in England and Wales	HIV Medicine	Qualitative	09
18	Woodford et al.	India	2015	Barriers and facilitators to voluntary HIV testing uptake among communities at high risk of HIV exposure in Chennai, India	Global Public Health	Qualitative	09
19	Zhao et al.	China	2018	Mhealth approach to promote oral HIV self-testing among men who have sex with men in China: a qualitative description	BMC Public Health	Qualitative	09
20	Iribarren et al.	USA and Puerto Rico	2020	Using an HIV self-test kit to test a partner: attitudes and preferences among high-risk populations	AIDS and Behavior	Mixed-method	09
21	John et al.	USA	2019	Gay and bisexual men’s experiences using self-testing kits for HIV and rectal and urethral bacterial sexually transmitted infections: lessons learned from a study with home-based testing	International Journal of Sexual Health	Mixed-method	09
22	Lipmann et al.	USA	2016	Acceptability and feasibility of HIV self-testing among transgender women in San Francisco: a mixed methods pilot study	AIDS and Behavior Springer	Mixed-method	09
23	Ong et al.	China	2018	Pressured HIV testing “in the name of love”: a mixed methods analysis of pressured HIV testing among men who have sex with men (MSM) in China	Journal of the International AIDS Society	Mixed-method	09
24	Reisner et al.	USA	2018	“Unspoken agreements”: perceived acceptability of couples HIV testing and counseling (CHTC) among cisgender men with transgender women partners	AIDS and Behavior	Mixed-method	09
25	Siegler	South Africa	2015	Exploring repeat HIV testing among men who have sex with men (MSM) in Cape Town and Port Elizabeth, South Africa	AIDS and Care	Mixed-method	09
26	Hart et al.	Nigeria	2017	Synergistic impact of sexual stigma and psychosocial well-being on HIV testing among Nigerian men who have sex with men: a mixed methods study	Gray literature	Mixed-method	09
27	Witzel et al.	UK	2016	What role does HIV self-testing (HIV-ST) have for men who have sex with men in the UK? Testing needs, social norms and biological citizenship	Gray literature	Qualitative	09
28	Witzel et al.	UK	2019	Pilot phase of an internet-based RCT of HIV-ST targeting MSM and transgender people in England and Wales: advertising strategies and acceptability of the intervention	BMC Infectious Diseases	Mixed-method	09
29	Daniels et al.	USA	2017	Getting HIV self-test kits into the home for young African American men who have sex with men in Los Angeles: a qualitative report	Journal of the Association of Nurses in AIDS Care	Qualitative	08
30	Freeman et al.	USA	2018	Perceptions of HIV self-testing among men who have sex with men in the United States: a qualitative analysis	AIDS Education and Prevention	Qualitative	08
31	Jaspal, R.	UK	2018	Perceptions of HIV testing venues among men who have sex with men in London and the Midlands, United Kingdom	Journal of Gay and Lesbian Social Services	Qualitative	08
32	Pal K., et al.	Cambodia	2016	acceptability study on HIV self-testing among transgender women, men who have sex with men, and female entertainment workers in Cambodia: a qualitative analysis	Plos One	Qualitative	08
33	Pharr JR, Lough NL, Ezeanolue EE.	USA	2015	Barriers to HIV testing among young men who have sex with men: experiences from Clark County, Nevada	Global Journal of Health Science	Qualitative	08
34	Tobin et al.	USA	2018	Acceptability and feasibility of a peer mentor program to train young Black men who have sex with men to promote HIV and sexually-transmitted infection home-testing to their social network members	AIDS and Care	Qualitative	08
35	Wayal et al	UK	2014	Home sampling kits for sexually transmitted infections: preferences and concerns of men who have sex with men	Culture, Health and Sexuality	Qualitative	08
36	Wei et al.	China	2018	Which user errors matter during HIV self-testing? A qualitative participant observation study of men who have sex with men in China	BMC Public Health	Qualitative	08
37	Rael et al.	USA	2020	Transgender women’s experiences using a home HIV testing kit for partner testing	AIDS and Behavior	Mixed-method	08
38	Reisen et al.	Colombia	2014	HIV testing among men who have sex with men in Bogotá, Colombia: the role of structural and individual characteristics	AIDS Education and Prevention	Mixed-method	08
39	Sullivan et al	USA	2015	Adaptation of the African couples HIV testing and counseling model for men who have sex with men in the United States: an application of the ADAPT-ITT framework	Springer Plus	Mixed-method	08
40	Flowers et al.	UK	2016	Preparedness for use of the rapid result HIV self-test by gay men and other men who have sex with men: a mixed methods exploratory study among MSM and those involved in HIV prevention and care	HIV Medicine	Mixed-method	08
41	Hines et al.	USA	2017	HIV testing and entry to care among trans women in Indiana	Journal of the Association of Nurses in AIDS Care	Qualitative	07
42	Nunn A., et al.	USA	2012	African American patient experiences with a rapid HIV testing program in an urban public clinic	Journal of the National Medical Association	Qualitative	07
43	Chen et al.	Australia	2010	Australian men who have sex with men prefer rapid oral HIV testing over conventional blood testing for HIV	International Journal of STD & AIDS	Mixed-method	07
44	Dirisu et al.	Nigeria	2018	Experiences with use of oral HIV self-testing (HIV-ST) among men who have sex with men and linkage to care: translating evidence to programmatic strategies for HIV-ST scale-up in Nigeria	Gray literature	Qualitative	07
45	Logie et al.	Jamaica	2016	Stigma and discrimination in HIV testing services: exploring experiences of young transgender women and men who have sex with men in Kingston, Jamaica	Gray literature	Qualitative	07
46	Dowson et al.	UK	2011	Why some men who have sex with men present late for HIV testing: a qualitative analysis	AIDS and Care	Qualitative	06
47	Balán et al.	USA	2019	SMARTtest: A smartphone app to facilitate HIV and syphilis self- and partner-testing, interpretation of results, and linkage to care	AIDS and Behavior	Mixed-method	06
48	Mullens et al.	Australia	2019	Point-of-care testing (POCT) for HIV/STI targeting men who have sex with men in regional Australia at community ‘beat’ locations	BMC Health Services Research	Mixed-method	06
49	Rawat et al.	India	2020	Motivators and barriers toward HIV self-testing among men who have sex with men in two Indian cities	Gray literature	Qualitative	06
50	Paige et al.	USA	2018	An intervention to teach young men who have sex with men and transgender women of color how to HIV self-test with a friend: lessons learned in project TRUST	Gray literature	Qualitative	05
51	Prost et al	UK	2007	‘‘There is such a thing as asking for trouble’’: taking rapid HIV testing to gay venues is fraught with challenges	Sex Transmission and Infection	Qualitative	05
52	Stephenson et al.	USA	2011	Attitudes towards couples-based HIV Testing among men who have sex with men in three US cities	AIDS and Behavior	Qualitative	05

Source: Developed by the authors

### Population and testing strategies analyzed

The reviewed studies analyzed the barriers or facilitators for HIV testing among MSM and TGW or the preferences for HIV testing type and the acceptability of new HIV testing strategies. Concerning self-testing, 34.6% (*n* = 18) of the studies analyzed the barriers or facilitators of self-testing for MSM, 15.4% (*n* = 8) for MSM and TGW, and 1.9% (*n* = 1) for TGW. Regarding conventional tests, 19.2% (*n* = 10) of the studies analyzed barriers or facilitators in MSM and TGW, 5.7% (*n* = 3) analyzed only MSM, and 3.8% (*n* = 2) analyzed only TGW. Regarding rapid testing, 7.7% (*n* = 4) of the studies analyzed only MSM, and 1.9% (*n* = 1) analyzed MSM and TGW. For HIV testing mixed methods, 3.8% (*n* = 2) analyzed the barriers and facilitators in MSM, 1.9% (*n* = 1) analyzed MSM and TGW and 3.8% (*n* = 2) explicitly analyzed barriers and facilitators without explaining the testing strategy.

### Methodological rigor

Of the 52 articles, 28 (53.8%) fulfilled at least nine of the items proposed by the CASP and were classified as having high methodological rigor, 21 (40.4%) fulfilled between six and eight items, and only three (5.8%) fulfilled 5 of the 10 items evaluated by the CASP.

### Social-ecological model: health promotion policies and programs vs. HIV testing strategies

In this category, we present evidence of HIV/AIDS programs that approach or distance themselves from the rationale of health promotion. A health promotion perspective, in this context, refers to the programs and policies to face the HIV/AIDS epidemic, addressing issues beyond the biomedical perspective, as proposed by the social-ecological model (Fig. 2).

Some studies have demonstrated that programs and policies can control the HIV/AIDS epidemic from a health promotion perspective. HIV testing in these contexts was presented as an all-encompassing rationale for different MSM and TGW populations with specific social needs, seeking to eliminate or minimize health inequities and barriers to HIV testing. Programs that incorporate community testing, community leader engagement, peer testing, social support networks, and strengthening of NGOs are particularly notable [17, 34–37].

Fauk et al. [24] stated that the Indonesian government has been committed to fighting the spread of HIV/AIDS through the establishment of policies and prevention programs, HIV testing, ART, and social support to improve individuals’ knowledge concerning HIV and enhance the existing testing programs.

Studies conducted with social networks in African American youth communities [38, 39] identified HIV self-testing as a potent strategy to increase the uptake of HIV testing among MSM and TGW. The authors indicated that, in addition to increasing HIV diagnoses, self-testing brings individuals closer to and facilitates their entry into clinical care and treatments such as ART.

Studies have also demonstrated the increasing incorporation of new HIV testing technologies, such as HIV self-testing, in the last decade. In total, 50% of the studies analyzed self-testing and the increasing use of information and communication technologies in health, mobile applications, and online social networks [40–46]. However, the incorporation of new testing technologies and communication strategies has not ensured access to HIV testing or information. For example, the prohibitive cost of HIV testing in some countries is considered a barrier to access.

### Vulnerability: understanding HIV testing outcomes

Using the theoretical framework of vulnerability, we analyzed the impact of the presence or absence of health promotion in HIV/AIDS programs and policies and how this interferes with positive or negative outcomes in HIV testing. Table 2 lists the present barriers and facilitators that interfere with HIV testing.

**Table 2:** Barriers and facilitators for different types of HIV testing at the individual, organizational, and community socio-economic levels

	**HIV Self-testing**			**HIV Conventional Test**			**HIV Rapid Test**		
	**Individual Level**	**Organizational Level**	**Community Level**	**Individual Level**	**Organizational Level**	**Community Level**	**Individual Level**	**Organizational Level**	**Community Level**
**Barriers**	- Feeling unable to perform the test -Technical challenges of performing the HIV test - Fear of pain or a positive result - Absence of a health professional - High cost of the test	- Absence of a professional in case of positive results - Bulky packaging involving self-tests - No Khmer language translation - Bureaucratic barriers of the health system in accessing free testing services	- Difficulty storing the self-test at home due to fear of parents finding out - Presence of conservative religions	- Low level of knowledge about services - Fear of a positive result - Low self-perceived risk	- Lack of confidence in the confidentiality of the results - Fear of discrimination from testing service professionals - Lack of professional cultural competence - Non-specific health policies without effective results	- Fear of discrimination from the community and family - Fear of being seen at a testing service and having your homosexuality revealed	- Fear of a positive result - Low self-perceived risk	- Fear of stigma and homophobia	No discussion
**Facilitators**	- Privacy - Convenience - Confidentiality	- Increased access to HIV testing without discrimination	- Support from community leaders for self-testing in the community - Presence of social support networks	- Understanding the benefits of knowing one's HIV status	- Establishing a bond with testing professionals - Knowledge of and easy access to testing sites - Professional and structural support to provide information and support - Test as part of the routine in some health services	- Social support from friends and partners - Open communication about HIV and sexual health with friends and partners	- Speed of receiving the result	- Good acceptability of the rapid test by the community	No discussion

Table 2 presents the barriers and facilitators to HIV testing in the three dimensions of the socio-ecological model, which are presented following the three dimensions of the theoretical model:

#### Individual dimension

MSM and TGW individuals' perceptions about HIV testing were identified in the individual dimension. The studies that evaluated individual’s relationship with the HIV test discussed the acceptability and usability of self-testing.

##### Barriers for HIV test: perceptions and feelings about HIV testing

The main barriers identified for the use of self-testing at the individual level were the technical challenges of performing the HIV test and the fear of a positive result without the immediate presence of a health professional (see Table 2). Additionally, the fear of a positive result and the prohibitive cost of the test were considered barriers to testing in the three types of tests: conventional, self-test, and rapid test [15, 18, 21, 23, 42, 47, 48].

Poor knowledge of testing services and insufficient understanding of the benefits of knowing one’s serological status were considered barriers to conventional testing and were related to low educational levels in some studies [18, 21, 24, 49]. Other studies showed that individuals with a higher level of education have doubts about the accuracy of the HIV self-test and fear a false negative result owing to the immunological window period of HIV infection [15, 19, 35, 39, 50].

##### Facilitators for HIV test

One study pointed out in addition to promoting self-test visibility and increasing user confidence, other strategies, such as providing tutorials on television or social networks on the correct self-test procedure, can be beneficial for target populations [43].

Studies discussing self-testing conveyed common facilitators, including convenience, confidentiality, and privacy, which encouraged individuals to engage in self-testing [19, 34, 38–41, 51–57]. Several studies have demonstrated that self-testing is an efficient strategy to increase the uptake of HIV testing among young MSM and TGW and, consequently, facilitate early diagnosis, care, and treatment [34, 38–41, 53–57].

#### Programmatic and organizational dimension

This dimension analyzed how health services are organized for HIV testing and the organizational structure of health systems and national and local HIV programs of the studies in question. The studies highlighted several structural and bureaucratic barriers to HIV testing and health services and systems, respectively.

##### Organizational structure of national and local HIV programs

Among all the studies analyzed, two published in India [16, 18] several individuals did not trust the confidentiality of the results, suffered discrimination from professionals in the field, and were threatened and harassed. Additionally, there was low acceptance of the testing service offered due to the absence of non-invasive testing methods (oral fluid self-testing), long queues, and poor physical facilities. They also verified funding policies preventing the hiring of peer counselors in specific contexts.

A study published in Colombia [21] highlighted bureaucratic barriers in the health system that hinder access to free testing services. According to the study, the HIV testing process is offered annually to MSM individuals and comprises four distinct stages: risk assessment and approval by a physician, pre-test consultation with a nurse or social worker, blood collection for testing, and post-test counseling. This study also notes that individuals needed to move to geographically distant locations at each stage of the testing process and bear displacement costs [21]. Difficulty in geographic access to testing services for individuals living in rural areas was also reported [21]. For example, a study conducted in Scotland reported difficulty in geographic access to HIV testing for individuals living in rural areas [58].

The presence of bulky packaging involving self-tests was reported to be a barrier as some individuals found it difficult to conceal the self-test inside their jeans pocket without being noticed, which would lead to questions about the nature of the package; for example, when walking on the street [47, 59]. Two Cambodian studies [42, 43] reported users’ difficulty in following the HIV self-test instructions because they were not translated into the local language.

##### New HIV testing strategies

Moreover, several studies have shown that unconventional testing strategies were well accepted or were a feasible alternative for offering HIV tests in certain specific contexts and in specific populations. For example, the provider-initiated testing and counseling (PITC) strategy proved effective in diagnosing early cases of HIV in MSM and TGW immigrants living in Spain; besides improving these individuals’ access to HIV testing, this strategy also minimized the stigma surrounding testing [60].

Like the PITC strategy, a mixed-method study conducted in New York in 2019 among Black and Hispanic or Latino MSM found that the implementation of the Opt-Out law testing as a routine to offer HIV testing in health services could increase the uptake of HIV testing and improve accessibility to HIV testing, and that the convenience of being tested in a routine consultation and knowing one’s HIV status stimulated preventive behaviors. However, approximately 30% of the individuals interviewed in the study reported feeling “threatened” or stigmatized by this testing law owing to concerns about the privacy and confidentiality of HIV test results [61].

Testing strategies conducted in mobile vans in Australia expanded the availability of HIV testing in various places and times and facilitated the scheduling of tests and the establishment of communication with individuals through social media. Conversely, difficulties were observed in adopting a good aesthetic for the vehicles to attract individuals to the vans for testing. Additionally, some researchers were concerned about the personal safety of individual volunteers who provided support and testing of HIV in their network of contacts and in the community (called “peer-testers”) during the night in isolated and unknown areas [62].

Some studies discussed the “partner testing” strategy [63–67] from two perspectives: testing couples who had steady partners and testing casual partners and sex workers’ clients. However, although couples with steady partners generally accepted this strategy, it was considered challenging for some TGWs with casual or transactional partners due to the possibility of violence against them, mainly because HIV testing was not acceptable for these partners [65, 66].

#### Social dimension: social support, community strategies and community leaders

Finally, the social dimension leads to community strategies for HIV testing. Several studies have described the strategies of distribution of peer tests through social support networks or testing in NGOs as important for increasing HIV testing rates, especially among young black MSM and TGW [24, 34, 35].

Social support among MSM peers plays a significant role in increasing the intention to participate in HIV testing services [24]. Confidence in social support networks is described as a facilitator; for example, for performing the HIV self-test [17]. Having social support from friends in the same social network during the HIV self-test was found to counterbalance the absence of professional counseling during and after the test and encourage individuals to regularly test themselves and seek health care [35].

Studies have also shown that the use of community strategies had positive results in the implementation of HIV testing services in culturally stigmatizing contexts, such as in some places in India and the Philippines [18, 43]. Thus, the main interventions for health promotion in relation to HIV testing based on the socio-ecological model are models of cultural changes in health, promotion of community health, initiatives of non-discriminatory public policies for strengthening NGOs, and community testing programs (see Fig. 2).

Regarding the availability of HIV tests and the role of community leaders, several studies have shown that bureaucratically institutionalized test arrangements in health institutions and services hindered access to HIV testing, whereas the participation of community services and NGOs and support from community leaders facilitated the use of self-testing and increased individuals’ confidence [21, 24, 34, 36–38, 61, 68].

## Discussion

Through the reviewed studies, the principal elements of three dimensions (i.e., individual, programmatic/organization, and social) were analyzed. The ecological model and the vulnerability framework, as well as some synergistic and complementary relationships between the dimensions were also examined. Furthermore, facilitators and barriers to HIV testing among MSM and TGW were identified in the three dimensions.

Some studies have shown that HIV testing services, which seek to logically promote health, operationalize HIV testing in a more comprehensive, inter-sectoral, and capillary way and a closer and more dialogue-driven manner with the community. In a larger discussion, Mol (2008) [69] addresses this kind of issue as the transposition of the “logic of choice,” centered on the provision of interventions and health technologies, to the “logic of care,” centered on the openness to knowing and discussing the concrete situations faced by people, as well on the refusal to reduce care to a “product” to be delivered. This transposition involves recognizing the coexistence of different “logics” that often generate contradictions and ambiguities in care practices and are commonly faced by people who hope to balance pleasure and risk control in sexual practices with a view to not only safeguard health and safety but also search for the meaning of life. As Vasconcelos et al. [70] explain, we should think of “HIV/STI prevention from the perspective of the logic of care, and thus, as a process that is not linear but dynamic, open, fluid, and erratic with multiple interactions and effects.” In this sense, it is vital to promote cultural changes in health systems, minimize barriers (both organizational and relational), act to reverse inequalities and inequities historically present in HIV testing [21, 24, 34, 36–38, 61, 68], and seek to balance power relations for the expansion of more dialogical institutional communicative health practices. It is good to remember that the paths opened by communicative technologies can contribute to this increase [69].

Discussing and operationalizing HIV testing “out of the box” in the health sector allows us to understand health promotion in its expanded conception of the health-disease process, incorporating community participation and the inter-sectoral nature of the actions performed by HIV testing programs. Thus, commitment to health equity and human rights is reaffirmed by recognizing the “collective rights of subjects” [69] that are shaped by the different “key populations” [72].

Conversely, the reviewed studies also identified that some health services and systems remain ill-prepared to perform HIV testing among MSM and TGW based on an expanded rationale for health promotion. Articles produced in India, Africa, Colombia, and Cambodia showed that the health systems of these countries still have organizational and structural deficits and bureaucratic barriers (technocratic actions that are rigid and insufficiently consider the perspectives of users and operate at the level of an “over the counter” health service) [71] with regard to HIV testing [16, 18, 21, 42, 43].

Accordingly, health systems need to address barriers in HIV testing and “rebuild” their national HIV programs to incorporate an expanded conception of health, strengthen human rights, create enabling environments free of discrimination and violence for MSM and TGW individuals, strengthen inter-sectoral and community approaches to HIV testing and individual autonomy, and encourage individuals to self-test.

Successful HIV prevention and awareness is a matter of enabling new HIV testing based on the recognition of the political power of communities and individuals involved in the decision-making processes. It is about expanding the conception of HIV testing beyond a prevention mechanism and combining it with changes in environments that provide autonomy and empowerment to subjects, thereby facilitating their access to and use of HIV testing. Thus, the greater the efforts made to improve the programs associated with existing social resources, the greater the chances of strengthening individuals in the face of the HIV epidemic and minimizing barriers to testing [27].

## Conclusion

Based on the findings of the studies and discussions presented above, we conclude that a “reconstruction” of HIV testing in its governmental, sectoral, and community contexts is necessary. It is a matter of situating the HIV test in the context of social interactions and expanding it to an inter-sectoral and community perspective within a broader view of health based on overcoming the traditional biomedical model rooted in health services, which reflects a testing process that includes political, programmatic, and socio-cultural aspects beyond one that is exact, bureaucratic, and regulated from a biomedical perspective.

We need greater democratization of HIV testing, minimization of barriers, ease of access to and use of tests, empowerment of individuals and communities, recognition of their rights, and guarantees of equity. HIV testing based on the health promotion model reinforces the idea that the need for individuals to respond to the transformation of practices is not limited only to the individual and private matrix but also extends to social subjects within the public sphere of social life.

Therefore, we reiterate that health promotion has the potential to contribute to a global change in testing services so that services incorporate equitable benchmarks that promote health, strengthen subjects in the face of epidemics, and recognize and support the political power of communities.

### Supplementary Information

**Additional file 1.** Reluctance to face a diagnosis of HIV, concerns about lack of privacy and confidentiality, and lack of support.

## Data Availability

The datasets used and/or analysed during the current study available from the corresponding author on reasonable request.

## Abbreviations

AIDS: Auto-immunodeficiency syndrome
ART: Anti-retroviral therapy
CASP: Critical Appraisal Skills Program
DeCS: Health sciences descriptors
EU: European Union
HIV: Human immunodeficiency virus
NGO: Non-governmental organization
MeSH: Medical subject headings
MSM: Men who have sex with men
PEP: Post-exposure prophylaxis
PITC: Provider-initiated testing and counseling
PLHIV: People living with HIV
PrEP: Pre-exposure prophylaxis
PRISMA-ScR: PRISMA Extension for Scoping Reviews
TGW: Transgender women

## References

[1] Joint United Nations Programme on HIV and AIDS (UNAIDS). Estatísticas mundiais sobre o HIV [online]. 2021. https://unaids.org.br/estatisticas/#:~:text=Pessoas%20vivendo%20com%20HIV&text=36%2C7%20milh%C3%B5es%20%5B32%2C,HIV%20eram%20mulheres%20e%20meninas. Accessed 31 Oct 2021.

[2] Organização Pan-Americana de Saúde (OPAS). Novos casos de infecção por HIV aumentaram mais de 20% na América Latina na última década [online]. 2021. https://www.paho.org/pt/noticias/30-11-2020-novos-casos-infeccao-por-hiv-aumentaram-mais-20-na-america-latina-na-ultima. Accessed 25 Nov 2021.

[3] Marques ALM, Sorrentino IS, Rodrigues JL, et al. The impact of COVID-19 on marginalized groups: the contribution of intersectionality as theoretical and political perspective. [preprint]. March 23, 2021. 10.1590/interface.200712

[4] Kerr LR, Mota RS, Kendall C (2013). HIV among MSM in a large middle-income country. AIDS.

[5] Almeida EL, Araújo GBS, Santos VA (2022). Adesão dos portadores do HIV/AIDS ao tratamento: fatores intervenientes. Revista Mineira de Enfermagem.

[6] Mesenburg MA, Wehrmeister FC, Silveira MF (2017). Teste de HIV solicitado e espontâneo: um estudo de base populacional com mulheres de uma cidade do Sul do Brasil. Cad Saúde Pública.

[7] Magno LM, Silva LA, Veras MA (2019). Estigma e discriminação relacionados à identidade de gênero e à vulnerabilidade ao HIV/aids entre mulheres transgênero: Revisão sistemática. Cad Saúde Pública.

[8] Bavintonn BR, Rodger AJ (2020). Undetectable viral load and HIV transmission dynamics on an individual and population level: where next in the global HIV response. Curr Opin Infect Dis.

[9] Joint United Nations Programme on HIV and AIDS (UNAIDS). Estados-membros das Nações Unidas adotam nova Declaração Política para enfrentar desigualdades e acabar com a AIDS [online]. 2021 https://unaids.org.br/2021/06/estados-membros-das-nacoes-unidas-adotam-nova-declaracao-politica-para-enfrentar-desigualdades-e-acabar-com-a-aids/. Accessed 5 Jan 2022.

[10] Fraser H, Borquez A, Stone J (2021). Overlapping Key populations and HIV transmission in Tijuana, Mexico: a modelling analysis of epidemic drivers. AIDS Behav.

[11] John SA, Cain D, Bradford-Rogers J (2019). Gay and bisexual men's experiences using self-testing kits for HIV and rectal and urethral bacterial sexually transmitted infections: lessons learned from a study with home-based testing. Int J Sexual Health.

[12] Sullivan PS, Stephenson RS, Grazter B (2014). Adaptation of the African couples HIV testing and counseling model for men who have sex with men in the United States: an application of ADAPT-ITT framework. Springer Plus.

[13] MacPherson P, Chawla A, Jones K (2011). Feasibility and acceptability of point of care HIV testing in community outreach and GUM drop-in services in the northwest of England: a programmatic evaluation. BMC Public Health.

[14] Witzel TC, Rodger AJ, Burns FM (2016). HIV self-testing among men who have sex with men (MSM) in the UK: a qualitative study of barriers and facilitators, intervention preferences and perceived impacts. PLoS ONE.

[15] Hines DD, Draucker CB, Habermann B (2017). HIV testing and entry to care among trans Women in Indiana. J Assoc of Nurses AIDS Care.

[16] Woodford MR, Chakrapani V, Newman PA (2015). Barriers and facilitators to voluntary HIV testing uptake among communities at high risk of exposure in Chennai. India Glob Public Health.

[17] Dass CD, Geerken MB, Bal M (2020). Reducing health disparities: key factors for successful implementation of social network testing with HIV self-tests among men who have sex with men a non-western migration background Netherlands. AIDS Care.

[18] Beattie TSH, Bhattacharjee P, Suresh M (2012). Personal, interpersonal and structural challenges to accessing HIV testing, treatment and care services among female sex workers, men who have sex with men and transgenders in Karnataka South India. J Epidemiol Commun Health.

[19] Bilardi JE, Walker S, Reader T (2013). Gay and bisexual men's view on rapid self-testing for HIV. AIDS and Behav.

[20] Longaray DA, Ribeiro PRC (2010). Discutindo a relação entre os marcadores sociais de gênero e a homossexualidade. Diásporas, Diversidades, Deslocamentos.

[21] Reisen CA, Zea MC, Bianchi FT (2014). HIV testing among MSM in Bogotá, Colombia: The role of structural and individual characteristics. AIDS Educ Prev.

[22] Buss PM (2000). Promoção da saúde e qualidade de vida. Ciência e Saúde Coletiva.

[23] Dowson L, Kober C, Perry N (2012). Why some MSM present late for HIV testing: a qualitative analysis. AIDS Care.

[24] Fauk NK, Sukmawati AS, Wardojo SSI (2018). The intention of men who have sex with men to participate in voluntary counseling and HIV testing, and access free condoms in Indonesia. Am J Mens Health.

[25] Buss PM. Uma introdução ao conceito de promoção da saúde. In: Czeresnia D e Freitas CM eds. Promoção da saúde, conceitos, reflexões, tendências. Rio de Janeiro: Editora Fiocruz 2009:19–42.

[26] Stokols D (1996). Translating social ecological Theory into guidelines for community health promotion. Am J Health Promot.

[27] Ayres JRCM, Junior IF, Calazans GJ, et al. O conceito de vulnerabilidade e as práticas de saúde: novas perspectivas e desafios. Czeresnia D e Freitas CM eds. Promoção da saúde, conceitos, reflexões, tendências. Rio de Janeiro: Editora Fiocruz 2009:121–144.

[28] Calazans GJ, Pinheiro TF, Ayres JRCM (2018). Vulnerabilidade programática e cuidado público: Panorama das políticas de prevenção do HIV e da aids voltadas para gays e outros HSH no Brasil. Sexualidad, Salud y Sociedad - Revista Latinoamericana.

[29] Tricco AC, Lillie E, Zarin W (2018). PRISMA Extension for scoping reviews (PRISMA-ScR): Checklist and Explanation. Ann Intern Med.

[30] Noblit GW, Hare RD (1988). Meta-ethnography: synthesizing qualitative studies.

[31] Sattar R, Lawton R, Panagioti M (2021). Meta-ethnography in health care research: a guide to using a meta-ethnographic approach for literature synthesis. BMC Health Serv Res.

[32] Carvalho AI. Determinantes sociais, econômicos e ambientais da saúde. In Fundação Oswaldo Cruz. A saúde no Brasil em 2030 - prospecção estratégica do sistema de saúde brasileiro: população e perfil sanitário. Rio de Janeiro: Fiocruz 2013:19–38.

[33] CASP. CASP: making sense of evidence. Public Health Resource Unit. U. Oxford. London 2006.

[34] Daniels J, Marlin R, Medline A (2018). Getting HIV self-test kits into the home for young African American MSM in Los Angeles: a qualitative report. J Ass Nurses AIDS Care.

[35] Freeman AE, Sullivan P, Higa D (2018). Perceptions of HIV self-testing among men who have sex with men in the United States: a qualitative analysis. AIDS Edu Prev.

[36] Jaspal R (2018). Perceptions of HIV testing venues among men who have sex with men in London and the Milands. United Kingdom J Gay Lesbian Soc.

[37] Tobin K, Edwards C, Flath N (2018). Acceptability and feasibility of a per mentor program to train young Black men who have sex with men to promote HIV and STI home-testing to their social network members. AIDS Care.

[38] Frye V, Wilton L, Hirshfield S (2015). Just Because it’s out there, people aren’t going to use it.’’ HIV self-testing among young, Black MSM, and transgender women. AIDS patient care STDs.

[39] Frye VWL, Hirshfield S, Chiasson LD (2018). Preferences for HIV test characteristics among young Black men who have sex with men (MSM) and transgender women: implications for consistent HIV testing. PLoS ONE.

[40] Gohil J, Baja ES, Reden T (2020). Is the Philippines ready for HIV self-testing?. BMC Public Health.

[41] Medline A, Daniels J, Marlin R (2017). HIV testing preferences among MSM members of an LGBT community organization in LOs Angeles. J Ass Nurses in AIDS Care.

[42] Okoboi S, Twimukye A, Lazarus O (2019). Acceptability, perceived reliability and challenges associated with distributing HIV self-test kits to young MSM in Uganda: a qualitative study. J Int AIDS Soc.

[43] Pal K, Ngin C, Tuot S (2016). Acceptability study on HIV self-testing among transgender women, men who have sex with men, and female entertainment workers in Cambodia: a qualitative analysis. PLoS ONE.

[44] Wei C, Yan L, Xiaoyou S (2018). Which user errors matter during HIV self-testing? A qualitative participant observation study of men who have sex with men (MSM) in China. BMC Public Health.

[45] Wirtz AL, Clouse E, Veronese V (2017). New HIV testing technologies in the context of a concentrated epidemic and evolving HIV prevention: qualitative research on HIV self-testing among men who have sex with men and transgender women in Yangon. Myanmar J Int AIDS Soc.

[46] Zhao Y, Zhu X, Pérez AE (2019). MHealth approach to promote oral HIV self-testing among men who have sex with men in China: a qualitative description. BMC Public Health.

[47] Liu F, Qin Y, Meng S (2019). HIV self-testing among men who have sex with men in China: a qualitative implementation research study. J Virus Erad.

[48] Siegler AJ (2015). Exploring repeat HIV testing among men who have sex with men in Cape Town and Port Elizabeth. South Africa AIDS Care.

[49] Pharr JR, Lough NL, Ezeanolue EE (2015). Barriers to HIV testing among young men who have sex with men (MSM): experiences from Clark County. Nevada Global J Health Sci.

[50] Balán IC, Rios JL, Nayak S (2019). SMARTest: A smartphone app to facilitate HIV and syphilis self-and partner-testing, interpretation of results, and linkage to care. AIDS and Behav.

[51] Dirisu OO, Tun W, Sekoni A, et al. Experiences with use of oral HIV self-testing (HIVST) among men who have sex with men (MSM) and linkage to care: translating evidence to programmatic strategies for HIVST scale-up in Nigeria. AIDS 2018, conference report (2018).

[52] Flowers P, Riddell J, Park C (2016). Preparedness for use of the rapid result HIV self-testing by gay men who have sex with men (MSM): a mixed-methods exploratory study among MSM and those involved in HIV prevention and care. HIV Med.

[53] Rawat S, Dange A, Shunmugam M, et al. Motivators and barriers toward HIV self-testing among men who have sex with men in two Indian cities [online] AIDS Conference (2020).

[54] Wray T, Chan PA, Simpanen E (2017). eTEST: Developing a smart home HIV testing kit that enables active, real-time follow-up and referral after testing. JMIR Mhealth Uhealth.

[55] Witzel TC, Bourne A, Burns FM (2019). HIV self-testing intervention experiences and kits usability: results from a qualitative study among men who have sex with men in the SELPHI (Self-Testing Public Health Intervention) randomized controlled trial in England and Wales. HIV Med.

[56] Witzel TC, Weatherburn P, Burns FM, et al. What role does HIV self-testing (HIV-ST) have for men who have sex with men (MSM) in the UK? Testing needs, social norms and biological citizenship. 21st International AIDS Conference (2016).

[57] Witzel TC, Gabriel MM, McCabe L (2019). Pilot phase of an internet-based RCT of HIVST targeting MSM and transgender people in England and Wales: advertising strategies and acceptability of the intervention. BMC Infect Dis.

[58] Boydel N, Buston K, McDaid LM (2017). Patterns of HIV testing practices among young gay and bisexual men living in Scotland: a qualitative study. BMC Public Health.

[59] Iribarren S, Lentz C, Sheinfil A (2020). Using an HIV self-testing kit to test a partner: attitudes and preferences among high-risk populations. AIDS Behav.

[60] Navaza B, Abarca B, Bisoffi F (2016). Provider-initiated HIV testing for migrants in Spain: a qualitative study with health care workers and foreign-born sexual minorities. PLoS ONE.

[61] Nanin L, Drumhiller K, Gaul Z (2019). HIV testing among black and Hispanic/Latino men who have sex with men in New York city: a mixed-method study. Arch Sex Behav.

[62] Mullens AB, Duyker J, Brownlow C (2019). Point-of-care testing (POCT) for HIV/STI targeting MSM in regional Australia at community "beat" locations. BMC Health Serv Res.

[63] Stephenson R, Sullivan PS, Salazar LF (2011). Attitudes towards couples-based HIV testing among MSM in three US cities. AIDS and Beha.

[64] Ong JJ, Wu D, Huang W (2018). Pressured HIV testing "in the name of love": a mixed methods analysis of pressured HIV testing among men who have sex with men in China. J Int AIDS Soc.

[65] Reisner SL, Menino D, Leung K (2018). Unspoken agreements": acceptability of couples HIV testing and counseling (CHCT) among cisgender men with transgender women partners. AIDS and Behav.

[66] Rael CT, Giguere R, Lopes-Rios J (2020). Transgender women's experiences using a home HIV-testing kit for partner testing. AIDS and Beha.

[67] Wayal S, Llewellyn C, Smith H (2011). Home sampling kits for sexually transmitted infections: preferences and concerns of men who have sex with men. Cult Health Sex.

[68] Paige MQ, Wilton L, Lucy C (2018). An intervention to teach young MSM and transgender women of color how to HIV self-test with a friend: lessons learned in project TRUST. AIDS 2018 Conference THPDC0106.

[69] Mol A. The logic of care health and the problem of patient choice. Taylor & Francis e-Library, 2008. Calazans GJ. Políticas públicas de saúde e reconhecimento: um estudo sobre prevenção da infecção pelo HIV para homens que fazem sexo com homens. Universidade de São Paulo (tese). 2018:223.

[70] Vasconcelos L (2023). Between risk and pleasure: reflections on HIV prevention and care in the current context of PrEP use by men who have sex with men. Cad Saúde Pública.

[71] Sader E (1988). Quando novos personagens entram em cena: experiências, falas e lutas dos trabalhadores da Grande São Paulo, 1970–80.

[72] Pires R, Lotta GS, Dutra R (2018). Burocracias Implementadoras e a (Re)Produção de Desigualdades Sociais: Perspectivas de Análise no Debate Internacional. In: Roberto Pires, Gabriela Lotta, Vanessa Elias de Oliveira. (Org.). Burocracia e Políticas Públicas no Brasil: Interseções Analíticas.

